# Abscess Formation after Septic Arthritis in the Sternoclavicular Joint of Two Healthy Men

**DOI:** 10.1155/2015/292854

**Published:** 2015-12-30

**Authors:** Jeppe Henriksen, Mariann Tang, Vibeke Hjortdal

**Affiliations:** Department of Cardiothoracic and Vascular Surgery, Aarhus University Hospital, Palle Juul-Jensens Boulevard 99, 8200 Aarhus N, Denmark

## Abstract

Abscess formation after septic arthritis in the sternoclavicular joint is a rare phenomenon in healthy people without immune suppression, intravenous drug abuse, or diabetes. Here we report two cases with formation of abscess in two middle-aged men, with no relevant comorbidities and no obvious sites of infection. The abscesses were both treated surgically with debridement followed by negative pressure wound therapy and antibiotics. The cases differ in diagnostic procedures and delay of diagnosis and broach the issues of handling a rare disease.

## 1. Introduction

Formation of an abscess after a septic arthritis in the sternoclavicular joint (SCJ) is a rare phenomenon among healthy people. Untreated, the abscess can have devastating consequences, why it is important to know the common symptoms and strategy of treatment. Here we report two cases of abscesses in the sternoclavicular joint.

## 2. Case Report

**Figure 1:**
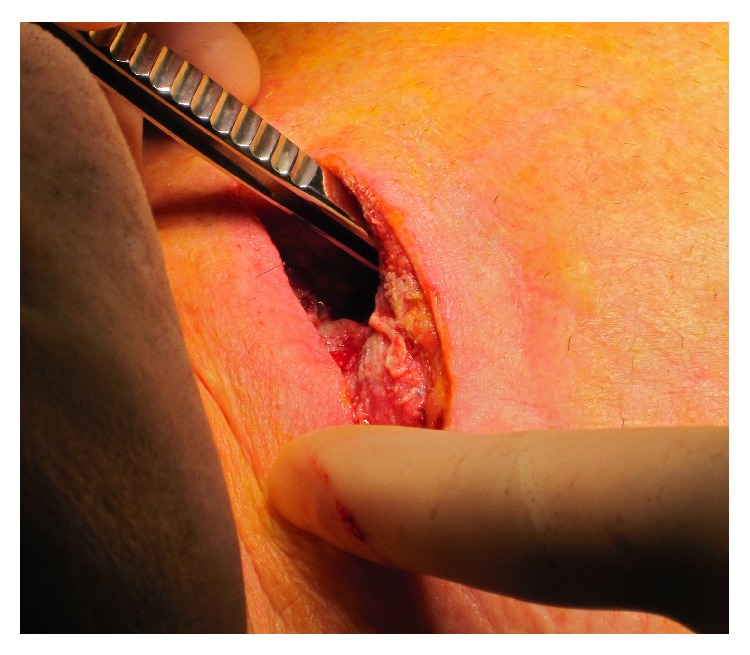
Patient 1. A view of the abscess cavity.

A 47-year-old man was admitted to the hospital with pain and immobilization of his right shoulder. The symptoms started suddenly and were accentuated over a few days with fever up to 39.0°C and redness and swelling above the SCJ. The C-reactive protein (CRP) was elevated 48 times (391 mg/L) above limit value at the admission. The patient was nondiabetic, was nonsmoking for five years, had no substance abuse, had no other infections, had no signs of immune deficiency, did not take immune suppressing medication, and was otherwise healthy. Blood cultures showed* Staphylococcus aureus*, and antibiotics were targeted. He was screened for endocarditis, urinary tract infection, and infectious skin lesions with negative results. A computed tomography (CT) scan showed inflammation in the joint and nearby tissue, and an abscess measuring 2,5 × 4 cm. Ten days after debut of symptoms he had surgical drainage and vacuum assisted closure therapy for 3 weeks (Figure 1). The joint was saved and no permanent damage was seen. Relevant antibiotics were maintained for 4 weeks.

**Figure 2:**
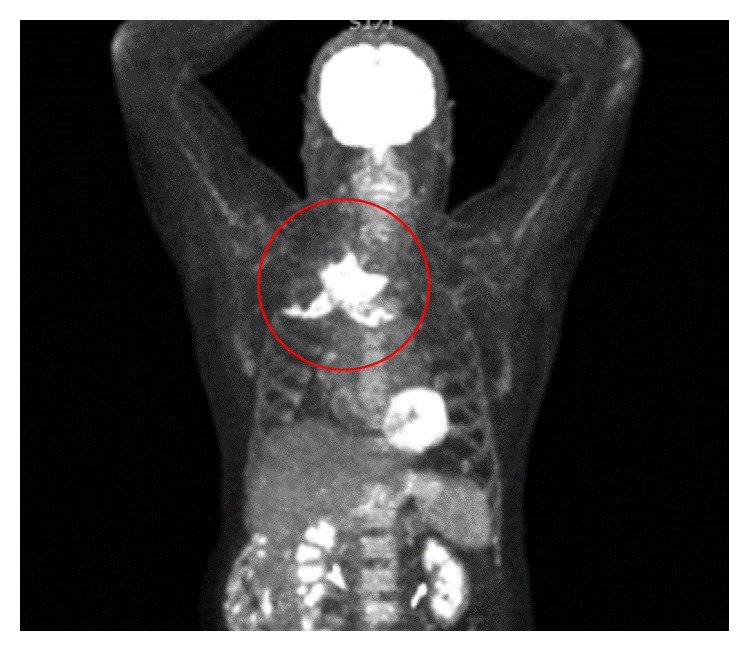
Patient 2. PET-CT frontal view, with reaction in the right sternoclavicular joint.

**Figure 3:**
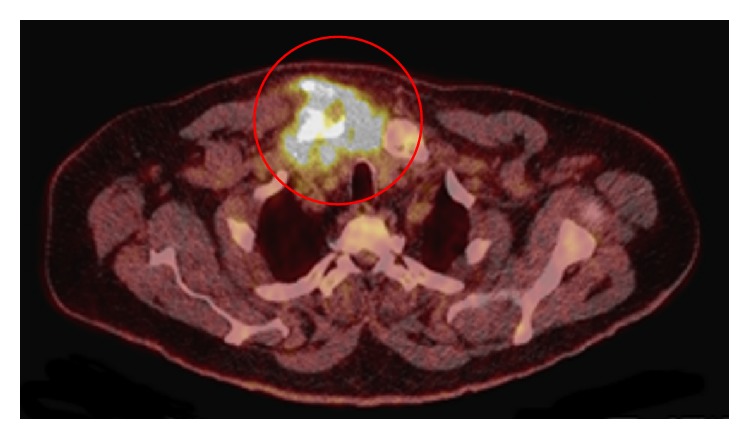
Patient 2. PET-CT cross-sectional view, with reaction in bone, joint, and nearby tissue.

The second case was a 64-year-old man presenting with pain from his right shoulder, left knee, and right ankle. The SCJ was swelled and sore. The CRP was elevated 17 times (141 mg/L) above value limit, and the temperature was 38.3°C at admission. The signs were misinterpreted as an attack of gout, since the patient had suffered from previous episodes with gout in ankle and knee-joint. The patient was nondiabetic, was a heavy smoker, had no substance abuse, had no signs of other infections, had no signs of immune deficiency, and did not take immune suppressing medication. The pain worsened and CRP increased over the next week and fluctuation above the SCJ appeared. Fluid aspiration from the knee and a urine sample both revealed* Staphylococcus aureus* infection, and relevant intravenous antibiotics were prescribed. Despite two weeks of antibiotic treatment, the symptoms continued. A CT scan showed inflammation in the SCJ and a light swelling in the nearby tissue. A puncture of the joint was attempted but failed to aspirate any pus, and a reactive arthritis was suspected instead of a septic arthritis. A positron emission tomography- (PET-) computed tomography after 36 days disclosed an infectious focus with formation of an abscess in the SCJ (Figures 2 and 3). The abscess measured 6 × 5 cm. The joint and head of the clavicle were eroded and the intercostal muscle was destroyed. The patient had surgical drainage. The cavity was treated with vacuum assisted closure for two weeks, and Aquacel for another two weeks along with four weeks of antibiotics. The infection vanished and the wound healed, but the SCJ is destroyed.

## 3. Discussion

Septic arthritis only affects the SCJ in 1% of the cases and is often misdiagnosed [1]. Septic arthritis in the SCJ is most commonly seen among intravenous drug abusers, immunosuppressed patients, or diabetics. Only a few cases among healthy persons have previously been reported. Abscess formation was found in 20% of 65 cases of septic arthritis in a review from 1988 [2]. The two cases presented here exemplify the importance of early diagnosis and timely intervention. Patient one was surgically treated within 10 days after onset of symptoms and was discharged without any permanent disability. Patient two was misdiagnosed at first, and the 36-day delay probably resulted in destruction of the joint. The delay, from the appearance of symptoms to diagnosis and treatment, is a general issue in arthritis of SCJ. Bodker et al. reviewed 10 cases, focusing on delay of treatment and radiologic examinations [3]. They found a frequent delay and the radiologic examination of choice to be magnetic resonance imaging (MRI). In our second case, a CT scan showed some reaction around of the joint, while a PET-CT visualized both abscess and bone affection.

It is believed that surgical drainage is the most effective treatment of an abscess. However, our knowledge is based on few cases and is not scientifically evident [4].

In conclusion, presence of pain, redness, or swelling of the SCJ calls for a CT or MRI investigation. Waiting for an antibiotic response is a potential delay of necessary surgical treatment. Awareness of possible abscess formation is important.
